# Multifunctional Nanopolymers for Blood–Brain Barrier Delivery and Inhibition of Glioblastoma Growth through EGFR/EGFRvIII, c-Myc, and PD-1

**DOI:** 10.3390/nano11112892

**Published:** 2021-10-28

**Authors:** Rameshwar Patil, Tao Sun, Mohammad Harun Rashid, Liron L. Israel, Arshia Ramesh, Saya Davani, Keith L. Black, Alexander V. Ljubimov, Eggehard Holler, Julia Y. Ljubimova

**Affiliations:** 1 Department of Neurosurgery, Cedars-Sinai Medical Center, Los Angeles, CA 90048, USA; Rameshwar.Patil@cshs.org (R.P.); Tao.Sun@cshs.org (T.S.); MohammadHarun.Rashid@cshs.org (M.H.R.); liron.israel@cshs.org (L.L.I.); Keith.Black@cshs.org (K.L.B.); Ljubimov@cshs.org (A.V.L.); 2 University of California Los Angeles, Los Angeles, CA 90095, USA; arshia6254@g.ucla.edu; 3 University of California Davis, Davis, CA 95616, USA; saya.davani@gmail.com; 4 Department of Biomedical Sciences, Cedars-Sinai Medical Center, Los Angeles, CA 90048, USA; 5 Department of Medicine, David Geffen School of Medicine, UCLA, Los Angeles, CA 90095, USA; 6 Terasaki Institute for Biomedical Innovation, Los Angeles, CA 90024, USA

**Keywords:** multifunctional drugs, blood–brain barrier, receptor-mediated transcytosis, brain tumor, delivery peptides, nanocarriers, cancer immunology, mRNA therapy

## Abstract

Glioblastoma (GBM) is the most prevalent primary brain cancer in the pediatric and adult population. It is known as an untreatable tumor in urgent need of new therapeutic approaches. The objective of this work was to develop multifunctional nanomedicines to treat GBM in clinical practice using combination therapy for several targets. We developed multifunctional nanopolymers (MNPs) based on a naturally derived biopolymer, poly(β-L-malic) acid, which are suitable for central nervous system (CNS) treatment. These MNPs contain several anticancer functional moieties with the capacity of crossing the blood–brain barrier (BBB), targeting GBM cells and suppressing two important molecular markers, tyrosine kinase transmembrane receptors EGFR/EGFRvIII and c-Myc nuclear transcription factor. The reproducible syntheses of MNPs where monoclonal antibodies are replaced with AP-2 peptide for effective BBB delivery were presented. The active anticancer inhibitors of mRNA/protein syntheses were Morpholino antisense oligonucleotides (AONs). Two ways of covalent AON-polymer attachments with and without disulfide bonds were explored. These MNPs bearing AONs to *EGFR*/*EGFRvIII* and *c-Myc*, as well as in a combination with the polymer-attached checkpoint inhibitor anti-PD-1 antibody, orchestrated a multi-pronged attack on intracranial mouse GBM to successfully block tumor growth and significantly increase survival of brain tumor-bearing animals.

## 1. Introduction

Glioblastoma (GBM), the deadliest brain cancer, has a very poor prognosis that has not improved in the past 35 years [1,2,3]. The current clinical therapeutic approaches require new drug creations based on the latest technologies and knowledge of the GBM biology. Recently, gliomas have been well-characterized by genomic and molecular marker analysis under The Cancer Genome Atlas (TCGA) project [4,5,6]. These studies have revealed significant GBM heterogeneity, which may necessitate the finding of new treatment targets and novel treatment strategies. The tumor microenvironment including immune cells is another important regulator of malignant growth, mediating tumor invasion and escape from immune surveillance [7]. It also serves as a niche for cancer stem cells (CSCs) that appear to convey tumor resistance to therapy and relapse development [7,8,9,10,11]. Additionally, brain vasculature provides the blood–brain barrier (BBB), restricting the access of many drugs, including antibodies, to brain tumors. Thus, an effective anti-GBM therapy requires multifunctional therapeutics with the ability to cross the BBB, block tumor-specific biomarkers, and normalize the tumor microenvironment, including the antitumor immune response. Such a niche for new drugs can be filled with nanodrug delivery systems that can combine several drugs on the same platform and avoid systemic toxicity by delivering the cargo directly to GBM cells.

We have previously engineered and successfully tested in preclinical models a number of potent nanodrugs to treat GBM that use a nontoxic poly(β-L-malic) acid (PMLA) carrier platform, cross the BBB by transcytosis, and deliver the payload to tumor cells. Our goal was to develop new and effective treatments of untreatable cancer, glioblastoma. To this end, we designed and tested novel multifunctional nanopolymers (MNPs) never-before-used for GBM treatment. They provided, for the first time, combined mRNA therapy to block the EGFR/EGFRvIII and c-Myc that are overexpressed in GBM, together with a checkpoint inhibitor antibody to programmed cell death protein 1 (PD-1). These MNPs were designed to achieve targeted delivery to the brain cancer cells and stimulate anticancer immunity. As an additional novelty, previously used tumor-targeting monoclonal antibodies (mAbs) were substituted by inexpensive peptides for effective therapeutics delivery through BBB.

For GBM treatment, we selected a peptide with BBB-crossing abilities, Angiopep-2 (AP-2). It binds to the endothelial cells’ (ECs) low-density lipoprotein receptor-related protein (LRP) 1 that switches from recycling to a transcytosis pathway in fully differentiated ECs [12]. Angiopeps are vectors for brain delivery that operate through the LRP transport system. Demeule et al. [13] designed 96 Kunitz domain-related peptides and evaluated their potential for transport through the BBB. AP-2 (TFFYGGSRGKRNNFKTEEY) was found to be the best in improving drug targeting to the brain [14,15]. So far, mostly small molecular drugs, e.g., paclitaxel-AP-2 [16] or nanoparticles with doxorubicin-AP-2, were used for BBB delivery [17], with modest effect. AP-2 is also useful for brain tumor targeting as LRP-1 is overexpressed in tumors [18]. AP-2-conjugated paclitaxel was used in a clinical trial against brain metastases of breast cancer [19]. We recently used a PMLA nanopolymer conjugated with AP-2, which significantly enhanced BBB penetration and the antitumor effect [20,21]. The PMLA as a nanoplatform allows one to covalently attach a number of anticancer moieties, e.g., antisense oligonucleotides (AONs) targeting c-Myc or EGFR/EGFRvIII, and checkpoint inhibitor antibodies such as anti-PD-1 (αPD-1), to produce a multifunctional nanomedicine against brain cancer.

c-Myc is known as a multifunctional activator of cell proliferation, DNA replication, protein biogenesis, global changes in cellular metabolism, the angiogenic switch, suppressor of the response to autocrine and paracrine regulatory programs, and a mediator of host immune responses. c-Myc activation appears to be a molecular hallmark of cancer, whereas partial suppression of c-Myc back to its physiological levels can result in sustained tumor regression and is associated with tumor cells’ proliferative arrest, differentiation, senescence, apoptosis, remodeling of the tumor microenvironment, mediation of immune response, and inhibition of angiogenesis [22,23]. In addition, the *c-Myc* oncogene is a cancer stem cell (CSC) biomarker. It was also recently shown to positively regulate the adaptive immune checkpoint PD-L1 (send critical “don’t find me” signal), as well as innate immune regulator CD47 (“don’t eat me” signal) [24]. The suppression of c-Myc in tumor cells caused a reduction in the levels of CD47 and PD-L1. c-Myc thus appears to initiate and maintain tumorigenesis, in part, by modulating immunoregulatory molecules [24]. c-Myc-mediated regulation of PD-L1 and CD47 was shown to be causally involved in the recruitment of T cells and macrophages [25,26,27]. c-Myc-driven tumors are likely to express high levels of immune checkpoints that suppress the antitumor immune response and, thereby, help evade immune surveillance. However, this oncogene’s action through immune checkpoints may also render relevant tumors highly susceptible to immunotherapy [28,29].

Recently, we found that epidermal growth factor receptor (EGFR) inhibition in intracranial glioblastoma multiforme (GBM) grown in two xenogeneic mouse models using a PMLA-based nanopolymer blocked the expression of c-Myc and other CSC markers, with a concomitant decrease in PD-L1, the ligand of PD-1 checkpoint [30]. This might not only induce cancer cell and CSC apoptosis and tumor growth arrest but also restore immune response to brain cancer cells [30].

Our comprehensive characterization of more than 500 glioblastoma tumors (GBMs) in the frame of The Cancer Genome Atlas (TCGA) Research Network identified mutated genes, as well as complex rearrangements of signature receptors, with most frequent changes including *EGFR* and *PDGFRA* [5,6].

EGFR is a receptor tyrosine kinase (RTK) overexpressed in 57% of human GBMs. RTKs are key regulators of growth factor signaling that controls cellular proliferation, metabolism, and survival [31]. Therefore, it is not surprising that alterations of RTKs including EGFR (that is, mutation, rearrangement, altered splicing, and/or focal amplification) have major roles in the development and progression of GBM [5]. A small number of GBM cells within a tumor seem to express both wild-type EGFR and EGFRvIII mutant proteins. In these cells, wild-type EGFR may phosphorylate EGFRvIII to activate downstream signaling. The EGFRvIII expression has been associated with a more aggressive tumor phenotype through paracrine mechanisms [22].

Polymeric drugs have a unique ability to put together a number of anticancer effectors on one platform for molecular combination therapy. The MNPs with multiple functional groups can be synthesized in a controlled way with high reproducibility. The MNPs used here traverse the BBB and deliver two different kinds of anticancer agents to the GBM cells: RNA therapeutics, that is, AON inhibitors of *EGFR*/*EGFRvIII* and *c-Myc* (regulators of many tumorigenic events and immunostimulators), or a checkpoint PD-1 inhibitor antibody (αPD-1) to prevent immune suppression. In order to function, AONs enter the cancer cell cytoplasm through the endosome escape mechanism, as we published previously [32,33]. We have also thoroughly confirmed the validity of the AP-2 peptide for tumor cell entry [21] and endosome membranolysis by the PMLA copolymer using the pH-sensitive LLL moiety [34].

## 2. Material and Methods

### 2.1. Reagents

Polymalic acid (PMLA) with molecular mass 60,000 D (SEC-HPLC/polystyrene sulfonate standards, polydispersity 1.2) was isolated from the culture supernatant of *Physarum polycephalum* M3CVII, as previously described [35,36]. Trileucine (H-Leu-Leu-Leu-OH) was from Bachem (Torrance, CA, USA). Mal-PEG3400-Mal and mPEG5000-NH2 were obtained from Laysan Bio (Arab, AL, USA). Rhodamine Red C2 maleimide was purchased from Thermo Fisher Scientific (Waltham, MA, USA). Superdex G-75 was obtained from GE Healthcare (Anaheim, CA, USA). In vivo MAb anti-mouse PD-1 (clone j43, Isotype Armenian hamster IgG) was from BioXCell (Lebanon, NH, USA).

### 2.2. GBM Cell Line

Mouse glioblastoma cell line GL261 was a gift from Dr. B. Badie’s lab (Beckman Research Institute, City of Hope, CA, USA). This line is positive for EGFR and c-Myc molecular biomarkers that were selected as GBM targets [37,38]. The GL261 cells were cultured in Dulbecco’s modified Eagle’s medium (DMEM; Thermo Fisher Scientific, Waltham, MA, USA) containing 10% fetal bovine serum with a 1% mixture of penicillin (100 u/mL), streptomycin (100 μg/mL), and amphotericin B (0.25 μg/mL) at 37 °C with 5% CO_2_. This cell line is not in the database of ICLAC’s commonly misidentified cell lines. Cells were routinely checked for mycoplasma (a kit from Lonza, Bend, OR, USA) with negative results.

### 2.3. Fluorescent Staining for BBB Permeation

Frozen brain tissue blocks prepared from drug-treated animals were sectioned at 7–10 μm using a Leica CM 3050S cryostat (Leica Microsystems, Buffalo Grove, IL, USA). Before staining, tissue sections were air-dried at room temperature (RT), fixed with ice-cold acetone for 10 min, rinsed three times with PBS, and mounted. Images were captured using a Leica DM6000B microscope (Leica Microsystems, Buffalo Grove, IL, USA). Direct fluorescence immunohistochemistry was used with labeled lectins.

The nanoconjugates with/without the attached AP-2 peptide for testing the MNPs’ delivery across the BBB were labeled with rhodamine (rh) P/LLL (40%)/rh and P/LLL (40%)/AP-2/rh and then injected intravenously (IV) 120 min before euthanasia to tumor-bearing animals at a concentration of 0.274 µmol/kg. Two lectins for brain vascular endothelium labeling were IV-delivered 15 min before mouse euthanasia. They comprised a mixture of 75 µL of 1 mg/mL Lycopersicon Esculentum (Tomato) lectin DyLight 488 and 50 µL of 5 mg/mL Ricinus Communis Agglutinin I (RCA I, RCA120) Fluorescein (both from Vector Laboratories, Burlingame, CA, USA). Three mice (*n* = 3) were used for BBB permeation imaging and staining experiments.

### 2.4. Intracranial Tumor Model and Treatment Regimen

All animal experiments complied with all relevant ethical regulations for animal testing and research and were performed under Cedars-Sinai Medical Center Institutional Animal Care and Use Committee (IACUC) approved protocol No. IACUC009043.

Twenty thousand GL261 cells in 2 μL of PBS were implanted intracranially into the right basal ganglia of immunocompetent 8-weeks-old female C57BL/6J mice (The Jackson Laboratory, Bar Harbor, ME, USA). All treatments were started on the third day after tumor cell inoculation. MNPs and controls were administered at doses of 10 mg/kg for each AON and ~10 mg/kg for αPD-1 via tail vein injections, twice per week for a total of 6 injections. The tumor-bearing mice were randomized into different groups for various drug treatments a day before the treatment started. Due to the use of several experimental and control drugs plus the standard control group, there was no possibility to perform a blinded treatment study in order to not mix the groups. However, imaging of BBB permeation was performed using animal numbers only by researchers blinded to a specific treatment group.

### 2.5. Prevention of Anaphylactic-Like Adverse Effects

Multiple treatments using nanodrugs with multiple moieties require an immune toxicity consideration, particularly when αPD-1 was used, due to its systemic toxicity. To prevent anaphylactic-like adverse effects, starting with the second treatment, all mice (including the control group) received 200 μg of antihistamine Triprolidine (Sigma-Aldrich, St. Louis, MO, USA) and 100 μg of platelet-activating factor (PAF) antagonist CV6209 (Santa Cruz Biotechnology, Dallas, TX, USA) via intraperitoneal injection, as we have published [21]. Briefly, Triprolidine and CV6209 were IP-administrated 30 min and 45 min, respectively, prior to the second to sixth injections of MNPs.

### 2.6. Synthesis of Novel Nanodrug Variants for Combination Brain Cancer Therapy

Synthesis of PMLA MNPs with high reproducibility and precision is critical and challenging for the nanomedicines with multiple functions and further in successful combination therapy against cancers.

**Figure 1:**
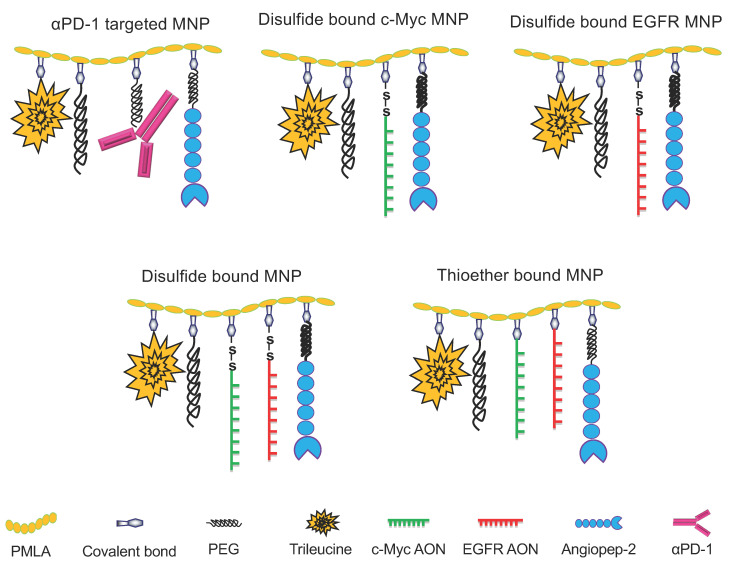
Schematic presentation of BBB crossing multifunctional nanoparticles (MNPs) to target c-Myc and EGFR, as well as αPD-1 MNP.

We achieved reproducible synthesis of various MNPs with controlled conjugation of each individual component [32,36,39] (Figure 1). The PMLA nanoconjugate was generally arranged for a two-step synthesis. First, a pre-conjugate containing mPEG5000-NH2, trileucine (LLL), and 2-mercaptoethylamine (MEA) was prepared on the PMLA backbone. Pre-conjugates can be lyophilized for long-term storage and conveniently used to prepare various functional nanoconjugates. The production of PMLA for MNPs synthesis from the myxomycete *Physarum polycephalum* using Bioreactor BIOSTAT^®^ Cplus (Sartorius, Bohemia, NY, USA) was published by us [35,40]. It was purified and characterized by NMR, and final products/intermediates by SE-HPLC and reversed-phase-HPLC, as we have published [40,41].

**Figure 2:**
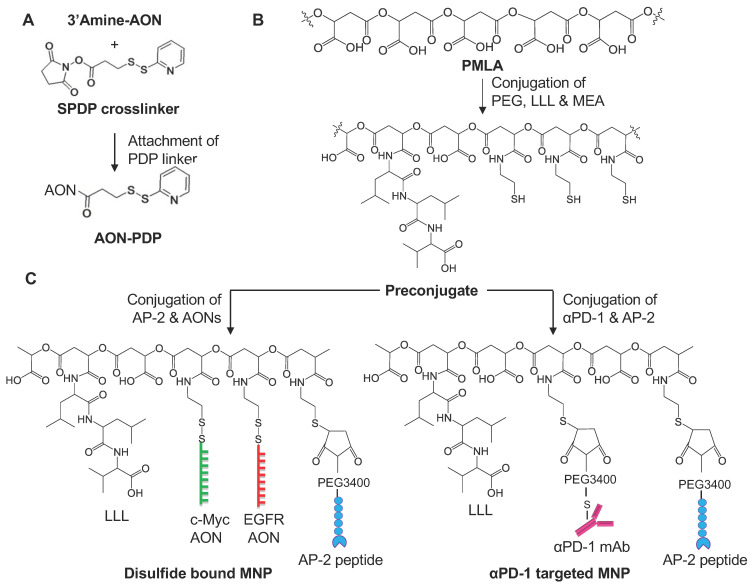
Synthesis of MNPs carrying disulfide bound (S-S) AONs to target c-Myc and EGFR, as well as αPD-1. (**A**) Synthesis of AON-PDP; (**B**) conjugation of PEG and LLL to PMLA (pre-conjugate); (**C**) synthesis of full MNPs.

The pre-conjugate P/LLL/MEA (Figure 2B,C) was synthesized in a one-pot reaction. PMLA was first activated with N-hydroxysuccinimide (NHS) in the presence of dicyclohexylcarbodiimide. mPEG3400-NH2 for protection and functional groups LLL and MEA were added sequentially after the completion of each amidation (Figure 2B). The reaction completion was confirmed by thin-layer chromatography (TLC; Ninhydrin test). The unreacted polymer-bound NHS group was decomposed in phosphate buffer pH 6.8. The pre-conjugate was purified on a PD-10 column, lyophilized, and stored at −20 °C.

### 2.7. Synthesis of Various Multifunctional Nanoparticles (MNPs)

Functional moieties were conjugated to the pre-conjugate to form MNPs: peptide for crossing the BBB and for tumor targeting, immune checkpoint inhibitor mAb for immunomodulation, and CTL activation and Morpholino AONs to block synthesis of cancer biomarkers c-Myc and EGFR/EFGRvIII to induce cancer growth arrest. Functional moieties were conjugated by reacting with the thiol group of the PMLA pre-conjugate through bifunctional linkers: maleimide-PEG5000-maleimide (Mal-PEG3400-Mal) for mAbs and peptides, and succinimidyl-3-(2-pyridyldithio)-propionate (SPDP) for AON. Before conjugating with the pre-conjugate, each functional group was activated with the bifunctional linker (Figure 2).

#### 2.7.1. Synthesis of 3-(2-Pyridyldithio) Propionyl AON (AON-PDP)

Morpholino AONs were conjugated to PMLA with a disulfide bond cleavable by disulfide exchange with cytosolic glutathione (GSH). Mouse AONs for *c-Myc* and *EGFR*/*EFGRvIII* (GeneTools, Philomath, OR, USA) were designed as follows: *c-Myc* 5′-CCAACGCCCAAAGGAAATCCAGCCT-3′ (25 nucleotides); and *EGFR*/*EGFRvIII* 5′-CTGAGGGTCGCATCTCTGACCG-3′ (22 nucleotides). 3′-Morpholino-NH_2_ residue was activated by the reaction of SPDP forming AON-PDP that is purified by acetone precipitation [42], lyophilized, and stored at −20 °C.

#### 2.7.2. Synthesis of S-Succinimidyl-PEG3400-Maleimide mAb

Susceptible αPD-1 mAb disulfide bonds in phosphate buffer were reduced with 5 mM of Tris(2-carboxy ethyl) phosphine hydrochloride (TCEP), purified on a PD-10 column, and conjugated with Mal-PEG3400-Mal followed by size-exclusion Sephadex G75 purification. Purified mAb(S-succinimidyl-PEG3400-maleimide) was concentrated by diafiltration (30 kDa cutoff). Successful conjugation of Mal-PEG3400-Mal to mAbs was verified by SEC-HPLC. The synthesized mAb-PEG3400-Mal was used the same day.

#### 2.7.3. Synthesis of S-Succinimidyl-PEG3400-Maleimide Peptide

**Figure 3:**
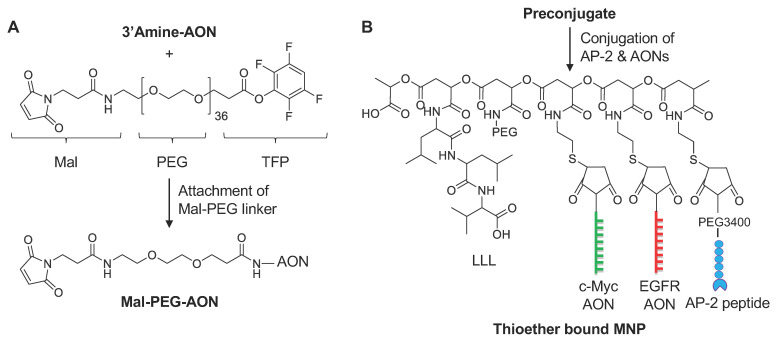
Synthesis of MNP carrying thioether-bound (direct) AONs to target *c-Myc* and *EGFR*. We used a thioether bond (direct conjugation) to attach AONs. The term “direct AON” was used to simplify AON attachment, differing from S-S-bound traditional synthesis, yielding cleavable AONs. (**A**) Synthesis of Mal-PEG-AON; (**B**) synthesis of full “direct AON” MNP.

For easy conjugation to the pre-conjugate, a BBB-crossing peptide AP-2 (TFFYGGSRGKRNNFKTEEYC) was used with an additional cysteine at the C-terminus for the introduction of the thiol group. It was conjugated with a single PEG as we have published [20]. It was added dropwise to maleimide-PEG3400-maleimide (molar ratio, 1:1.05) in phosphate buffer pH 6.3. This “click” reaction finished within 30 min. The resultant AP-2-PEG3400-Mal was used directly for conjugation with the pre-conjugate. The final MNPs, see full list in Table 1, were synthesized as shown on Figure 2. Pre-conjugate P/mPEG5000/LLL/MEA in 100 mM of phosphate buffer pH 6.3 was added to the mixture of, e.g., αPD-1-PEG3400-Mal, in the same buffer at RT, resulting in the desired stoichiometry (1 or 2 mAb molecules per PMLA chain). Then, AP-2-PEG3400-Mal was similarly conjugated to PMLA, but with higher loading (2%) to increase binding efficacy. Complete mAb and AP-2 conjugation was verified by SEC-HPLC that should yield a single product peak. In the case of MNP carrying AONs (either single or combination), P/mPEG5000/LLL/AP-2/MEA was added to AON-PDP to conjugate AON. Final products (Figure 2, Table 1) were purified on a Sephadex G75 column. As the synthesis of MNPs carrying disulfide-bound AONs suffers from low conjugation efficiency/yields (generally <25%), we developed a synthetic method to attach AONs via a stable thioether bond. The major advantage of this strategy is that the conjugation efficiency is very high (>90%). This also allowed us to control the number of AONs on each MNP with much better control/precision (Figure 3, Table 1). AONs are among one of the most expensive materials and take up major resources for the synthesis.

### 2.8. General Procedure for Synthesis of Mal-PEG-AONs

To a solution of 3′amine-modified AON (30 mg, 3.55 µmol), dissolved in a mixture of 1.5 mL of DMF and 0.5 mL of PBS, MAL-PEG-TFP (14 mg, 7.1 µmol) was added, and the reaction mixture was stirred at RT for 1.5 h. The reaction was monitored by SEC-HPLC and completed within 1.5 h. Acetone (10 mL) was added to the reaction mixture and the precipitate was centrifuged at 3000× *g* for 5 min. Clear solution was discarded, and the precipitate was resuspended in 10 mL of acetone and centrifuged again at 3000× *g* for 5 min. Clear acetone solution was discarded and the product was dissolved in water and lyophilized to obtain a whitish solid.

### 2.9. Physicochemical Characterization of MNPs. Synthesis Monitoring

Each batch of the pre-conjugate was verified for pH-sensitive endosomolytic delivery using liposome leakage assay [20]. Successful mAb and AON conjugation was monitored by SEC-HPLC following retention time and UV absorbance at 220, 260, and 280 nm. For the synthesis of new MNPs, each addition of new components was monitored with an Alpha FT-IR spectrometer (Bruker, Billerica, MA, USA) for the formation of new bonds and the disappearance of the old bonds [35,39]. The size and ζ-potential of each MNP were determined (Table 2) in solution in Zetasizer Nano-ZS90 (Malvern Panalytical, Malvern, UK). Individual components were quantified in solution. Total malic acid was assessed with a malate dehydrogenase assay after complete nanodrug hydrolysis [43]. mAb and AON content was quantified by our new method for the simultaneous determination of mAb or peptides (e.g., AP-2):AON, after selective cleavage of the PMLA backbone with NH_4_OH [44]; and PEG, by ferrothiocyanate extraction [39,45]. By ester cleavage of the PMLA backbone, each component, mAb, AON, and AP-2 (or AON/peptide), can be separated by size-exclusion HPLC, and all components were identifiable by a DAD detector. Absolute molecular weight determination was made with SEC-HPLC in line with our multi-angle light scattering (MALS) detector (DAWN HELEOS) and refractive index detector (OptiLab rEX, Wyatt Technology, Santa Barbara, CA, USA). MALS determined the absolute molecular weight of MNPs and their size, shape, and conformation in solution [46]. With these techniques, MNPs were characterized and their synthesis can be improved based on available structural data. In quality controls, the activity of AON release and the amount of AON binding to MNPs were assessed by a rapid assay with GSH- or DTT-dependent release, as described [21,47,48] (Table 1).

### 2.10. Statistical Analysis

Statistical analysis of survival data was carried out using Kaplan–Meier curves and the log-rank test using Prism 8 program (GraphPad Software, San Diego, CA, USA). *p* < 0.05 was considered statistically significant.

## 3. Results

### 3.1. In Vivo Study of BBB-Crossing Ability of Used MNPs

**Figure 4:**
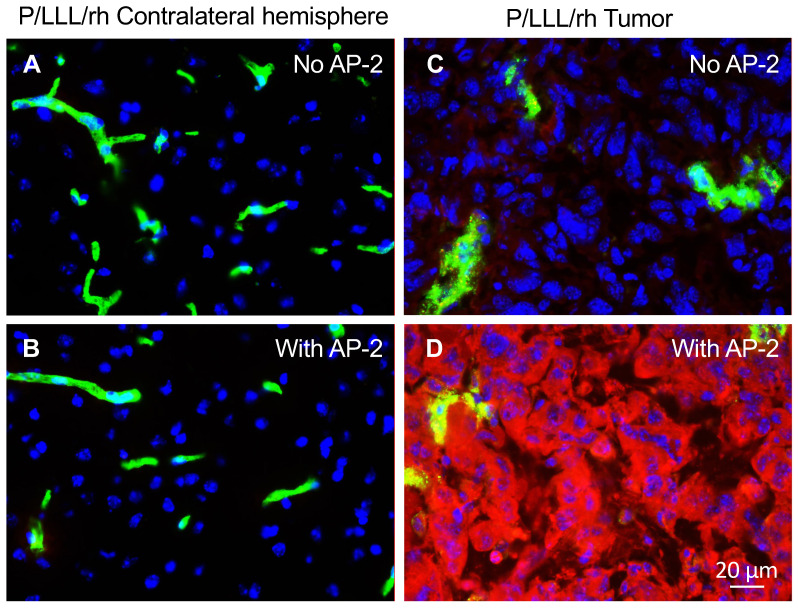
AP-2 enables BBB crossing by MNPs after I.V. injection. (**A**) P/LLL/Rhodamine in unaffected brain. (**B**) P/AP-2/LLL/Rhodamine in unaffected brain. No red signal is visible. (**C**) P/LLL/Rhodamine in GL261 GBM. Very weak red signal. (**D**) P/AP-2/LLL/Rhodamine in GL261 GBM, showing strong signal in the tumor cells. MNPs with the PMLA backbone, AP-2 peptide, LLL, and rhodamine were IV-injected into mice with intracranial GL261 GBM. Animals were sacrificed 3 h post-drug injection. In addition, 15 min before mice were euthanized, lectin-AF488 (green color) was injected IV to label blood vessels. Frozen sections of mouse brains were imaged in a Leica DM6000 B system (scale bar = 20 µm). Blue: DAPI (nuclear); green: lectin-AF488 (blood vessels); red: rhodamine (PMLA polymer).

The conjugates of P/LLL (40%), the BBB delivery vectors with AP-2 peptide, were previously shown to achieve high brain permeation [20,49]. Here, we wanted to obtain evidence of the BBB delivery using the syngeneic mouse model. The experiments were conducted using C57BL/6J (BL/6) mice with intracranial GL261 GBM-injected IV with the carrier-peptide conjugates and the controls and were euthanized 120 min post-injection. Brain sections photographed with a 40× objective are shown in Figure 4. They display a red fluorescence of the vector conjugates as they penetrate the brain parenchyma. We compared here how the conjugates with and without AP-2 peptide (P/LLL (40%) and P/LLL (40%)-AP-2) penetrate nontumor tissue (unaffected collateral hemisphere) and the tumor. The data demonstrated that a healthy, nontumor brain is not permeable for P/LLL (40%) or the P/LLL (40%)-AP-2 conjugate (Figure 4A,B). However, the BBB in the tumor even without AP-2 was slightly leaky for P/LLL (40%), producing a weak signal in the parenchyma Figure 4C. At the same time, the P/LLL (40%)-AP-2 (Figure 4D) yielded a very strong accumulation in the tumor parenchyma outside of the brain vessels (compare Figure 4C,D). These results confirmed the possibility of using P/LLL (40%)/AP-2 as a vector for the delivery of cancer molecular inhibitors directly to the tumors. They also suggested that the AP-2 peptide seems to have dual functions for delivery through the BBB and targeting brain cancer cells.

### 3.2. Brain Tumor Treatment In Vivo

The mouse glioblastoma GL-261 cell line at 2.0 × 10^4^ cells per mouse was stereotactically inoculated into mouse brain. Mice-bearing brain tumors were treated with MNPs injected IV twice a week, starting on day 3 post-tumor inoculation for a total of six treatments. All animals were followed for survival post-treatment. PBS as a drug solvent was used as a negative control and administered the same way as MNPs to a separate group of mice.

The PBS control group and six experimental groups of mice (*n* = 8/group; one mouse from group 4 died after injection 5 from surgical complication and was removed from the study) bearing intracranial GL-261 GBM were systemically treated with MNPs (Table 2). Group 1 is a group where the MNP with the αPD-1 checkpoint inhibitor mAb was used as a GBM suppressor, as we published [21], and group 2 represents mRNA therapeutics, anti-mouse AON against wild and mutated *EGFR*/*EGFRvIII* (one oligonucleotide was designed to suppress both gene variants) and anti-mouse AON against *c-Myc*. All other groups 3–6 represent the treatment where the mRNA therapeutics were combined with another MNP having a covalently attached αPD-1 IgG1 mAb (Figure 2 and Figure 3). Group 6 was treated with a MNP containing an uncleavable thioether bond to test whether cytoplasmic AON release from the PMLA through a cleavable S-S bond was needed for the drug action. Animals in all groups were premedicated with 200 μg of antihistamine Triprolidine (Sigma-Aldrich, St. Louis, MO, USA) and 100 μg of platelet-activating factor (PAF) antagonist CV6209 (Santa Cruz Biotechnology, Dallas, TX, USA) via intraperitoneal injection, as we published [21], to prevent immunotoxicity such as cytokine storm/anaphylaxis. The premedication was performed before second MNP injection and followed the other four drug IV administrations.

**Figure 5:**
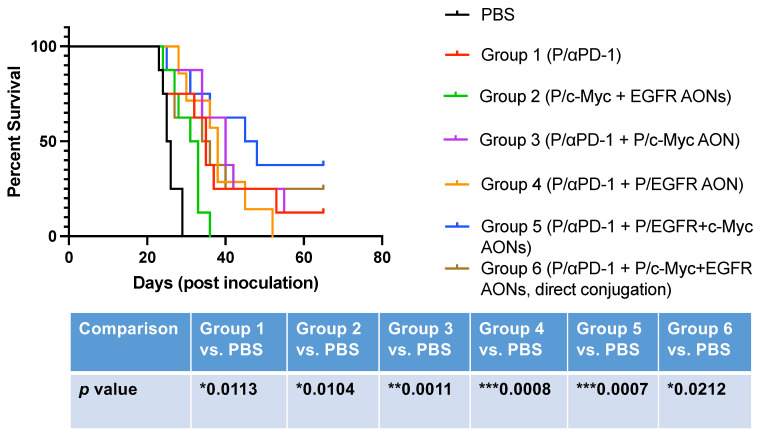
Survival of mice with intracranial GL261 GBM after various MNP treatments. All groups showed significant effect vs. PBS control. The strongest beneficial effects were observed in combination groups, where the inhibition of GBM markers by MNPs was associated with administration of an immunostimulatory checkpoint inhibitor (groups 3–5). “Direct AON” conjugation produced a significant but modest effect suggesting that, for full efficacy, AONs should be cleaved off the PMLA backbone. Combination treatment groups 3–5 were significantly different from PBS and single treatment group 1.

The survival in all groups was significant for all experimental groups 1–6 compared to PBS-treated group. However, the combination treatment in groups 3–5 yielded the best survival that was significantly different from PBS or group 1 (Figure 5). Group 6, where AONs were “directly” attached to the PMLA nanoplatform, is different from group 5 (Table 2), because the AON bond with PMLA was not cleavable by cytoplasmic GSH, unlike disulfide bond -S-S- linker for AONs in all other groups. As we described before, we wanted to simplify and standardize the MNPs production. Directly attached AONs allowed us to control the number of AONs on each MNP with much better precision. It could also reduce the cost of the drugs, because AONs are very expensive materials and take up major resources for the synthesis. Although the survival effect in group 6 was modest, it was still significant (Figure 5) and appears to be fully acceptable for the treatment, so this MNP can be potentially used for in vivo brain cancer treatment. Moreover, two mice from this group were in the category of long survivors for 65 days.

## 4. Discussion

The unique CNS environment provides limited treatment options, in part because of inefficient drug delivery across the BBB [50,51]. Conventional therapies to treat GBM in the clinic have not resulted in an improvement of patient survival. Understanding the biological mechanisms that are implicated in mutations found in gliomas, and the role of the tumor microenvironment and immunosuppression, is vital in designing novel classes of interventions that are safe, efficacious, and can provide a lasting response. Combining targetable therapy with immune checkpoint inhibitors seems to yield better clinical outcomes and survival benefits. Developing appropriate preclinical models in the setting of brain cancer [52] is also a necessity for the advancement of novel therapies that can be translated to clinical practice. Currently, a number of clinical trials where immunotherapy alone was used by engaging major approaches such as oncolytic vaccines, chimeric antigen receptor-expressing T cells (CAR-T), and checkpoint inhibitors [53] for glioma have not yielded significant improvements. This outcome concerns 28 clinical trials for vaccines (e.g., a peptide vaccine that targets EGFRvIII or IDH1), 13 clinical trials for oncolytic viruses, 15 phase III clinical trials for checkpoint inhibitor mAbs (e.g., CheckMate 143 trial), and CAR-T cells, none of which have surpassed the efficacy of GBM standard-of-care, that is, temozolomide/radiation therapy, with 14.6 months of average survival [53]. Although CTLA-4 and PD-1 checkpoint inhibitor mAbs do not cross the BBB [54,55], a small efficacy against GBM was still found in animal studies, which could result from general immune system activation.

Recently, an effective preclinical treatment of GBM was developed using a novel combination treatment approach. The molecular therapy by activating glucocorticoid-induced TNFR-related receptor (GITR) in Treg cells reprograms them in order to reduce the resistance of immune checkpoint Treg cells to immunotherapy. When the activation of GITR was combined with the αPD-1 checkpoint inhibitor, the survival of glioma-bearing mice was significantly longer in comparison with monotherapy [56].

The role of immune checkpoint inhibitors in metastatic brain tumor treatment was recently reviewed. The authors concluded that metastatic tumors to the brain show significant immunosuppression that should be counteracted by combinations of biological drugs that could potentiate the effects of radiation, chemotherapy, and/or neurosurgery to provide efficient treatment of brain metastases [57].

Here, we combined for the first time a promising nanosystem based on a nontoxic natural-derived PMLA platform with covalently attached multiple AP-2 peptides for superior BBB delivery of mRNA inhibitors against *c-Myc* and *EGFR*/*EGFRvIII* to treat GBM, in combination with the checkpoint inhibitor, αPD-1 mAb, to achieve maximum treatment efficacy. It is also necessary that such a platform should be biodegradable to exclude initial systemic toxicity. Furthermore, the nanomedicines to treat central nervous system tumors have to be able to cross the BBB and lack neurotoxicity.

The MNPs synthesis and characterization are standard in our laboratory, which has a history for translational production of nano-immunodrugs [21,32,35]. Due to the polydisperse and polyvalent nature of the polymeric pre-conjugate, we tried to reduce the polydispersity by molecular sieving and improving the uniformity of final products. We standardized the synthesis of activated components and their conjugation with pre-conjugates. By fluorescence immunohistological analyses, we showed that PMLA-LLL (40%)/AP-2 efficiently crossed the BBB and accumulated in the tumor but not in a normal brain (Figure 4A–D), making AP-2 the lead BBB and tumor-targeting moiety. To deliver nanomedicines through the BBB for treating brain cancer, a number of parameters should be met such as optimal size, slight hydrophobicity, lack of toxicity and targetability, and lack of drug leakage in circulation [20,34,50,51,54,58]. Our MNPs are in the range of 9–15 nm in size and slightly hydrophobic, which is good for BBB crossing. They are nontoxic and are able to specifically target brain cancer cells, which makes them superior compared to other targeted nanoconstructs [20,50,51,54,58].

We prepared two classes of MNPs, with “indirect” synthesis through a cleavable disulfide -S-S- bond and “direct” attachment of mRNA therapeutics to PMLA. The direct synthesis was performed for the first time and significantly simplified the production of the MNPs preparation with more precise control, a higher yield of conjugates, and as a consequence, lower cost. The “direct” MNP showed inferior efficacy in animal survival compared to “indirect” MNPs, suggesting that AONs should be free from the platform to exert a maximum inhibitory effect on the target protein synthesis, possibly by removing steric or charge hindrance by PMLA. At the same time, “direct” MNP also showed a significant survival advantage, making it potentially usable for treatment due to simpler synthesis and low cost.

Combination therapy approaches have always been more effective to treat cancer than monotherapies. Along this line, new classes of drugs should be able to provide a multipronged attack on the tumor and have all necessary pharmaceutical parameters for successfully treating brain cancer. They have to target specific cells, pass through biological barriers, suppress the growth of tumor cells and regenerative precursors, such as cancer stem cells and angiogenic precursors, as well as increase antitumor reactivity of the immune system.

Recently, we developed nanotechnology for the delivery of checkpoint inhibitors αPD-1 and αCTLA-4 to the brain tumor to activate the brain local immune system [21]. Here, we further combined this approach by co-delivering PMLA/AP-2-attached αPD-1 with MNPs, inhibiting most often-expressed GBM markers c-Myc and EGFR/EGFRvIII [1,4,5,6,7]. We found that the suppression of c-Myc and EGFR/EGFRvIII significantly prolonged the survival of mice with orthotopic brain cancer (Figure 5). Long-term survival was observed in treated groups where these markers were targeted (up to 65 days), and the number of such mice was increased in combination treatment groups with αPD-1 MNP. Further studies using larger groups of animals may confirm our hypothesis about the effectiveness and the possibility of production of multifunctional nanopolymers with several tasks as a proof of principle for successful GBM treatment in the future.

Overall, we designed novel multifunctional nanomedicines that might be used to treat brain cancer. They showed effective BBB delivery, and they significantly prolonged brain tumor-bearing animal survival by the simultaneous inhibition of two major brain cancer markers, c-Myc and EGFR/EGFRvIII, that are present in a number of mutation-bearing GBM variants [1,4,5,6,7], with concomitant suppression of an immune checkpoint to boost tumor immune surveillance.

## Figures and Tables

**Table 1:** Summary of MNPs, their abbreviations, and physicochemical characterization.

Multifunctional Nano Polymers (MNPs)	Abbreviation	Hydrodynamic Diameter (nm)	ζ Potential (mV)
PMLA/PEG5000(2%)/LLL(40%)/ AP-2(2%)/αPD-1(0.2%)	αPD-1 MNP	15.2 (±1.7)	−9.5 (±0.4)
PMLA/PEG5000(2%)/LLL(40%)/ AP-2(2%)/c-Myc+EGFR AON(2.0%)	AON-SS MNP	8.9 (±0.9)	−9.2 (±0.8)
PMLA/PEG5000(2%)/LLL(40%)/ AP-2(2%)/c-Myc+EGFR AON-thioether(2%)	AON-Thioether MNP	10.6 (±0.9)	−10.1 (±0.7)
PMLA/PEG5000(2%)/LLL(40%)/ AP-2(2%)/c-Myc AON(2.0%)	c-Myc-AON MNP	9.6 (±0.9)	−8.3 (±0.5)
PMLA/PEG5000(2%)/LLL(40%)/ AP-2(2%)/EGFR AON(2.0%)	EGFR-AON MNP	9.3 (±1.1)	−9.4 (±0.9)

**Table 2:** List of MNPs used for syngeneic mouse treatment.

Group	MNP Composition
1	P/mPEG5000(2%)/LLL(40%)/AP-2*(2%)/αPD-1**(0.2%)
2	P/mPEG5000(2%)/LLL(40%/AP-2(2%)/EGFR/EGFRvIII AON***(2%)/c-Myc AON***(2%)
3	P/mPEG5000(2%)/LLL(40%)/AP-2(2%)/αPD-1(0.2%) co-administered with P/mPEG5000(2%)/LLL(40%)/AP-2(2%)/c-Myc AON(2%)
4	P/mPEG5000(2%)/LLL(40%)/AP-2(2%)/αPD-1(0.2%) co-administered with P/mPEG5000(2%)/LLL(40%)/AP-2(2%)/αPD-1 (0.2%)/EGFR/EGFRvIII AON(2%)
5	P/mPEG5000(2%)/LLL(40%)/AP-2(2%)/αPD-1(0.2%) co-administered with P/mPEG5000(2%)/LLL(40%/AP-2(2%)/EGFR/EGFRvIII AON(2%)/c-Myc AON(2%)
6	P/mPEG5000(2%)/LLL(40%)/AP-2(2%)/αPD-1(0.2%) co-administered with P/mPEG5000(2%)/LLL(40%/AP-2(2%)/EGFR/EGFRvIII AON****(2%)/ c-Myc AON****(2%)

MNPs for mouse treatment have a general structure of P/mPEG5000/LLL/AP-2/checkpoint inhibitor mAb or AONs. In some experiments, two MNPs were co-administered: * targeting molecules are selected to have dual functions for both tumor targeting and BBB crossing such as AP-2 peptide; ** checkpoint mAb, αPD-1; *** AONs are to mouse *c-Myc* and *EGFR*/*EGFRIII* chains where AONs were attached to PMLA with a disulfide bond; **** AONs are to mouse *c-Myc* and *EGFR*/*EGFRIII* where AONs were attached to PMLA with a thioether bond; PBS was used as a negative control.

## Data Availability

All data are available upon request.
